# Alcohol as a Dressing for Wounds, with Special Reference to Its Employment by M. Nèlaton, at the Clinical Hospital, Paris, France

**Published:** 1866-11

**Authors:** W. Lockwood Bradley

**Affiliations:** New Haven


*Alcohol as a Dressing for Wounds, with Special Reference to its Employment by M. Nèlaton, at the Clinical Hospital, Paris, France.* By W. Lockwood Bradley, M. D., New Haven.

[Read before the New Haven County Meeting, April, 1866.]

While State legislatures have been endeavoring by laws of prohibition and license to restrict the use of alcohol as a beverage, its employment as a medicinal agent has steadily increased. As it has been well expressed by Dr. Wilks, editor of the London *Lancet*, "there are few things more remarkable in the recent history of medicine than the extent to which alcohol has been introduced, and the importance which has been attached to it in the treatment of disease."

In the present paper, it is proposed to offer some remarks upon the treatment of wounds, surgical and traumatic, with alcohol. The idea thus enunciated is by no means original; on the contrary, it was known to Hippocrates, Galen, and Ambrose Parè. An analysis of preparations, employed by them in the treatment of wounds, need not be very searching to prove that alcohol was often the basis of their interminable and now superannuated formulas. Two illustrations will be sufficient. The "balsam of Fioraventi" was composed of turpentine, myrrh, aloes, ginger, canella, with other substances, and more than three thousand parts of alcohol. Again: the barbarous practice of scalding gunshot wounds with boiling oil was changed by Ambrose Paré for the employment, with other applications, of the alcoholic solution or tincture of myrrh.

Coming down to more modern times, alcohol was used by Baron Larry during the campaigns of the first Napoleon. Since then it has been employed as a popular remedy both in France and America. In the year 1859, M. Batailhé, of Paris, published ("De l'alcool et des composè alcooliques en chirurgie") the result of some experiments performed upon the lower animals. Among other points, it was proven that alcohol favors the immediate union of wounds in three ways: first, by arresting hemorrhage from the smaller vessels (blood being a great obstacle to perfect coaptation); second, by producing immediate coagulation of albumen; lastly, by promoting the plastic secretion.

At a somewhat later period, alcohol was introduced as a surgical dressing in two of the principal hospitals of Paris, by M. Dolbeau, at the St. Louis Hospital, and M. Nélaton, at the Clinical Hospital. Of forty-eight cases treated at the latter hospital during the first eight months of 1864, forty-two healed rapidly, and three after an attack of erysipelas. Three terminated fatally: one from cancer, one from phthisis, and one from purulent absorption. Thirty-nine were the result of important operations, such as amputation of the leg and the removal of an enormous tumor. In one case the denuded surface measured six inches in the transverse diameter. The record for 1863 shows an equal degree of freedom from pyæmia, erysipelas, and like accidents. M. Nélaton and others believe that these results are dependent, in a great measure, upon the therapeutical effects of alcohol.

I propose to consider these effects with special reference to changes which may take place in wounds healing by the second intention. For an accurate and scientific description of these changes, I am indebted to Mr. Paget. ("Lectures on Surgical Pathology," Philadelphia, 1860.) He informs us that after the infliction of an open incised wound, the blood gradually ceases to flow, and is followed by a blood-tinged or serous-looking fluid; this gradually becomes paler, and collects like a whitish film or glazing upon the surface of the wound. Moisture, whether in the form of water, dilute alcohol, or glycerine, will produce this result.

According to the same authority, this condition, called by him the state of calm or inactivity, is ended in from two to eleven days by the return of blood to the part. In what way the ordinary water dressing can favor this return it is difficult to understand. On the contrary, alcohol assists reaction, not only by its known power as a local excitant, but also by being absorbed, and thus stimulating the general circulation.

M. Chédervergne, of Paris, has published ("Du traitment des plaies chururgicales et trumatiques par les pausements a l'alcool, p. 15, 1864,) three cases going to prove and illustrate the truth of this statement. He states that in December, 1863, a patient entered the Clinical Hospital, carrying an enormous tumor, situated upon the posterior part of the left leg; this was removed, leaving a large denuded surface, extending from the propliteal space to the heel. An alcoholic dressing was applied, and five days after the patient showed symptoms of intoxication, which it was impossible to attribute to any other cause than the treatment employed. He also mentions (ibid p. 21,) two other cases in which a feeling of exhilaration was excited.

Looking at the subject merely from a theoretical point of view, we should fear that alcohol, by its local and constitutional effect, would excite excessive reaction. Such, however, is not the result of actual experiment at the Clinical Hospital; and M. LeCourt, professor at the Medical School of Caen, states ("Une lettre avec des observations cliniques sur l'emploi des alcooliques en chirurgie,". Paris, 1859) that he has employed alcohol as an application to wounds in at least fifty cases, and that in only a small proportion of the entire number was he compelled to suspend the use of the dressing on account of too great inflammatory action. Some degree of inflammation seems necessary, since, in the opinion of Mr. Paget, (p. 140,) the ordinary process of granulations in its commencement, morbid, and resembles inflammation in at least two points, namely: 1st, that the increased quantity of blood in the part producing granulations moves more slowly than in health; and 2dly, that the increased supply of blood precedes the increased production of material. This material is similar, in every visible respect, to coagulable lymph. If undisturbed, it will soon present minute points of vascularity; these gradually increase in extent, and in two or three days give place to granulations.

It is at this stage of the healing process we so often observe the inefficiency of water or cerate. The circulation in the part is so languid, that the granulations frequently become large, flabby, and livid. On the contrary, when alcohol is employed, the granulations are uniformly florid, granular, and scarcely raised above the surrounding tissues. Suppuration or degeneration of the plastic lymph is hardly perceptible.

M. Nélaton and others do not claim for alcohol an infallibility which does not belong to quinine or any other so-called specific; on the other hand, they *do* believe in its prophylactic power against pyæmia and erysipelas, and in confirmation of their belief, bring forward facts relating to the non-occurrence or diminished frequency of these affections. To appreciate the full meaning of these observations, we must remember that they were not collated from private practice, but in one of the largest hospitals of Paris—a hospital situated in one of the most unhealthy districts of the Latin Quarter, and presenting an unusual array of circumstances predisposing to surgical complications. Among such we may enumerate, crowding, poor ventilation, insufficient or inappropriate food, absence of the consolations and encouragements of friends, and generally constitutions naturally weak or debilitated by disease.

All of these influences were present in the surgical wards of the Clinical Hospital, and yet, under the employment of alcohol, only one case of pyæmia occurred during the first eight months of 1864. In like manner, during the first five months of the same year, there was not a single case of traumatic erysipelas, although numerous cases of an epidemic nature happened in other Parisian hospitals. About the first of June, however, the Interne of M. Nélaton reported three cases, of which the following is an abbreviated translation:

Observation 1st. The first case was that of a young man, aged sixteen years. He submitted to an operation for the removal of a large ganglion, situated in the region occupied by the parotid gland. General symptoms of erysipelas set in, with chill, fever, and derangement of the stomach. The wound looked well; but on the third day of the fever, an erysipelatous inflammation was discovered, occupying the shoulders, the scalp, and the eyebrows; in other words, surrounding the parotidean region, but always respecting the borders of the wound and the parts bathed with alcohol.

Observation 2d. The second case was that of a woman, aged sixty-two. On the 23d day of May, 1864, she underwent an operation for the removal of a cancerous tumor of the breast. The same general phenomena, as in the first case, showed themselves; in two days erysipelas appeared, with its customary character, upon the trunk, then upon the arm, near to the breast which had been removed, but did not invade the part which had been dressed with alcohol.

Observation 3d. The third case was that of a woman forty-nine years old. On the 24th day of May, she sustained an operation for the removal of a tumor on the thigh. The wound was dressed with alcohol, and for a time all went well; suddenly the appetite diminished, and a febrile reaction supervened, followed by a red œdematous inflammation upon the back. In three days this disappeared; but after seven days there was erysipelas of the face.

The three observations thus presented possess an interest even greater than those relating to the non-occurrence of erysipelas; they show the enemy no longer kept in the background, but actually making the attack and suffering defeat. In other words, they picture an erysipelatous inflammation spreading to the very precincts of the wound, and there being arrested.

In addition to what has already been said, it may be remarked, that alcohol, in common with water, surpasses all other applications in point of cleanliness. When first applied to a denuded surface, it causes a sensation of heat, but in a few minutes this disappears, and after two or three applications, on successive days, does not return. Patients, questioned upon this point, do not complain so much of the hot as of the cold sensation occasionally experienced.

A stranger, entering for the first time the surgical wards of the Clinical Hospital, will notice that the atmosphere is unvitiated by any foul odor, and unchilled by evaporating water.

And now a few words upon the best method of applying alcoholic dressings. The alcohol employed at the Clinical Hospital is about equal in strength to the dilute alcohol of the U. S. Dispensatory. Generally it contains a proportion of camphor; but this is not considered essential. Occasionally circumstances may require that the alcohol should be further diluted. We may raise the temperature of the mixture, and so avoid the disagreeable sensation of coldness by taking the strong officinal alcohol, and just before applying it, adding an equal volume of water. The preparation may be brought in contact with the wound by lint or oakum, and the evaporation be prevented, in a great measure, by thick cloth or oiled silk. Unusually it is sufficient to renew the dressing once or twice in the twenty-four hours.

In conclusion, I would say, that I have purposely avoided the discussion of theoretical points. Nor have I noticed an opinion which I once heard expressed by M. Maissonneuve, in effect, that alcohol causes paralysis of the blood vessels, and so predisposes to secondary hemorrhage. This theory was first proposed by Claude Bernard, to account for the non-absorption of a certain poison; and, so far as I am aware, its truth has never been demonstrated. It has rather been my object to present facts which have been clinically observed by the Surgeons and Internes of the Hôpital des Cliniques. It now remains with clinical students of this State and America to substantiate or subvert the foregoing conclusions; to determine how far the beneficial action of alcohol, as a surgical dressing, will warrant its substitution for less expensive and time-honored applications.—*From Proceedings of Connecticut Medical Society*, 1866.

